# A sterile plant culture system of *Uncaria rhynchophylla* as a biosynthetic model of monoterpenoid indole alkaloids

**DOI:** 10.5511/plantbiotechnology.25.0218a

**Published:** 2025-06-25

**Authors:** Takako Sugahara, Ryosuke Sugiyama, Hiroshi Sudo, Yuta Koseki, Katsuyuki Aoki, Mami Yamazaki

**Affiliations:** 1 Graduate School of Pharmaceutical Sciences, Chiba University; 2 Tsumura Botanical Raw Materials Research Laboratories, Tsumura and Co. Ltd.; 3 Plant Molecular Science Center, Chiba University; 4 Faculty of Pharmaceutical Sciences, Hoshi University

**Keywords:** biosynthesis, metabolome, monoterpenoid indole alkaloid, sterile culture, *Uncaria rhynchophylla*

## Abstract

*Uncaria* plants, belonging to the Rubiaceae family, develop characteristic hooks at their leaf axils. In the Japanese Pharmacopoeia, the hooks from three *Uncaria* species, including *U. rhynchophylla*, are collectively defined as “Uncaria Hook” and are widely used as medicinal materials. The pharmacological properties of the diverse bioactive metabolites in *U. rhynchophylla*, particularly monoterpenoid indole alkaloids (MIAs), have been extensively studied. In this study, we aimed to establish sterile cultures of *U. rhynchophylla* as models for investigating MIA biosynthesis. LC-MS/MS-based untargeted metabolomic analysis revealed that the metabolomic profiles of stems from cultured plants showed strong similarity to those of medicinal parts from mature plants, specifically the hooks and stems. Furthermore, the analysis indicated that the contents of oxindole and indole alkaloids exhibited distinct variations depending on the plant part and developmental stage, both in sterile plant cultures and mature plants. Our findings demonstrate that *U. rhynchophylla* can be maintained under sterile conditions while stably producing MIAs. These cultured plants can serve as a model system not only for studying MIA biosynthetic pathways but also for ensuring quality control of Uncaria Hook in medicinal applications. This model system would contribute to the fundamental research by enhancing our understanding of the biosynthetic mechanisms and facilitating applications such as metabolic control of the contents of bioactive compounds in Uncaria Hook.

## Introduction

The genus *Uncaria*, belonging to the family Rubiaceae, currently comprises 38 confirmed species (WFO Plant List [version June 2024, https://wfoplantlist.org/ (Accessed Oct 1, 2024)]). Plants in this genus are characterized by hooks at their leaf axils, which serve as a distinguishing feature (Wan et al. 2023). In the Japanese Pharmacopoeia, hooks from three *Uncaria* species—*U. rhynchophylla* Miquel, *U. sinensis* Havid, and *U. macrophylla* Wallich—are specified as crude drugs collectively known as “Uncaria Hook”. In China, these three species, along with *U. hirsuta* Havil and *U. sessilifructus* Roxb, are designated as Gou-Teng. Hooks and the surrounding stem branches are used as raw materials for medicinal products.

Previous studies have explored the morphological and chemical differences among *Uncaria* species (Pan et al. 2020; Sakakibara et al. 1999; Zhang et al. 2020). Among these species, *U. rhynchophylla*, distributed in Japan (southwards from Chiba Prefecture) and the central to southern parts of China, has garnered particular attention owing to its high abundance of bioactive metabolites, including monoterpenoid indole alkaloids (MIAs). MIAs are widely distributed in the families Rubiaceae, Apocynaceae, and Loganiaceae and are characterized by a spiro-carbon center linking an indole ring with a monoterpene moiety. These compounds exhibit potent biological activities; for example, vinblastine and its related metabolites possess anticancer properties (van der Heijden et al. 2004), while strychnine is a natural neurotoxin (Hong et al. 2022).

MIAs from *Uncaria* species were originally isolated in earlier studies (Aimi et al. 1982; Koseki et al. 2025; Li et al. 2017; Ma et al. 2009). Owing to their pharmacological importance (Huang et al. 2021; Li et al. 2023), the Japanese Pharmacopoeia mandates that the total alkaloid content (rhynchophylline and hirsutine) in dried Uncaria Hook must not be less than 0.03%. In *U. rhynchophylla*, MIAs are considered to be synthesized from strictosidine, a common precursor in MIA biosynthesis (Guo et al. 2014; Hu et al. 2024; Yang et al. 2022). However, the biosynthetic mechanisms underlying these processes remain poorly understood. The MIA content in *Uncaria* plants varies depending on the plant part (Qu et al. 2012) and environmental factors such as light intensity (Shao et al. 2024; Wang et al. 2022), ethylene response (Li et al. 2022), and soil composition (Liu et al. 2024). These metabolic variations provide valuable insights into MIA biosynthetic pathways. While analytical methods for these alkaloids are well-established (Wu et al. 2015; Yomura et al. 2004), studies on their distribution across different plant parts and developmental stages remain limited.

Sterile in vitro culture systems are an effective tool for metabolic research, facilitating the elucidation of biosynthetic pathways for various specialized metabolites. These systems enable the production of large quantities of genetically uniform plants within a short time under controlled growth conditions. In *Uncaria* plants, the establishment of tissue culture methods for *U. rhynchophylla*, including clonal propagation and acclimatization of plantlets, have been explored in breeding studies (Ishii et al. 2012, 2013; Taniguchi and Ishii 2015). Additionally, quantitative assays of metabolites, including MIAs, have been performed on clonal *U. rhynchophylla* plantlets (Yamamoto et al. 2023).

In this study, we established a sterile culture system for *U. rhynchophylla* and its metabolic profile, with a focus on alkaloid content. This study aimed to evaluate the potential of sterile plant cultures as a model for studying MIA biosynthesis and for quality control of Uncaria Hook in medicinal applications.

## Materials and methods

### Chemicals and reagents

The authentic standards were purchased from the following suppliers and used without further purification; rhynchophylline (RC) and hirsutine (HTI) from FUJIFILM Wako Pure Chemical; isorhynchophylline (IRC) from Toronto Research Chem; corynoxeine (CX), isocorynoxeine (ICX), corynoxine B, corynoxine, hirsuteine (HTE), and geissoschizine methyl ether (GME) from Med Chem Express. The standards of strictosidine, 5β-carboxystrictosidine, vincoside lactam, strictosamide, corynantheine, cadambine, and 3α-dihydrocadambine were isolated and purified from Uncaria Hook. All other solvents and reagents were obtained from commercial suppliers and used without further purification.

### Plant materials

Analytical samples of field-grown mature plants of *U. rhynchophylla* were collected from two trees in Kamogawa City, Chiba Prefecture, Japan. Seeds were harvested in November 2022, and aerial parts were collected from the same trees in July 2024. Voucher specimens (THS 110225, 110226) were deposited in the herbarium of Tsumura & Co., Ltd., Japan.

### Growth conditions

The fruits of *U. rhynchophylla* were air-dried at 40°C overnight, and the seeds were extracted using a mortar and pestle. The extracted seed fragments were purified using a sieve. The seeds were surface-sterilized by shaking them in 1–2% sodium hypochlorite for 10–15 min. After that, they were rinsed with sterilized water three times and sown onto φ90 mm petri dishes containing solid medium composed of half-strength Murashige and Skoog inorganic salts, half-strength B5 vitamins, 1.5% sucrose, and 0.8% agar. After two months of cultivation, the seedlings were transferred into φ40 mm glass tubes. The plants were transferred to new media according to their growth. The in vitro plants were maintained at 25°C under a 16-hour photoperiod with fluorescent lighting (50 µmol m^−2^ s^−1^).

### Sample preparation

#### Sampling

Sterile plant materials were obtained from in vitro plants. The seedlings of 1 and 2 months after germination were collected as whole plants (*n*=6 and 10). For the plants of 3–10 months after germination were separated into leaf, stem, and root tissues (*n*=2–9). For the plants of 10 and 12 months after germination (*n*=6), tissues were further divided into shoot apex, leaf, stem, and root. Leaves and stems at each node were sequentially numbered from the apical end of the plant stem and analyzed individually. The aerial parts of mature plants were separated into five tissues: stem tip, leaf, stem, hook, and immature fruit. Leaves, stems and hooks at each node were sequentially numbered from the apical end of the plant stem and analyzed individually. All plant materials were snap-frozen and lyophilized.

#### Metabolite extraction

One to eight mg of dried plant materials was placed into a 2.0 ml tube containing φ2.3 mm ceramic beads. The sample was pulverized for 5 min at 30 Hz using a TissueLyser II (QIAGEN). An extraction solution (80% methanol with 0.1% formic acid) was added at a concentration of 4 mg dry weight (dwt) ml^−1^. The tube was shaken at 10 Hz for 20 min using the same TissueLyser II to extract metabolites. After centrifugation at 11,700×g for 5 min, 10–100 µl of the supernatant was transferred into a new 2.0 ml tube and dried using a centrifugal evaporator. The residue was dissolved in a 10-fold volume of the extraction solution containing 1 mM lidocaine as an internal standard, resulting in a final concentration of 0.4 mg dwt ml^−1^. The solution was filtered through Ultra-Free MC hydrophilic PTFE (Merck Millipore) filters and subjected to LC-MS analysis. Blank samples were prepared using the same procedure without plant material.

### LC-MS/MS conditions

Standard solutions and extract solutions (10 µl) were subjected to LC-MS/MS analyses using the Vanquish ultra-high-performance liquid chromatography (UPLC) system equipped with an orbitrap mass spectrometer Exploris 240 (Thermo Fisher Scientific). Metabolites in the extract solution were separated using a ZORBAX RRHD Eclipse Plus C18 column (1.8 µm, 2.1 mm×100 mm, Agilent Technologies). Solvent A consisted of water with 0.1% (v/v) formic acid while solvent B consisted of acetonitrile with 10% (v/v) water and 0.1% (v/v) formic acid. The LC gradient program was set as follows: 0–0.5 min, 5% B; 0.5–10 min, 30% B; 10–17 min, 99.5% B; 17–18.5 min, 99.5% B; 18.5–18.6 min, 5% B; 18.6–20 min, 5% B; flow rate was set as follows: 0–17 min, 0.6 ml/min; 17–18.5 min, 1 ml/min; 18.5–20 min, 0.6 ml/min. The column oven was maintained at 40°C. The mass spectra were obtained by electrospray ionization under the following parameters: heated capillary temperature at 350°C; nitrogen sheath gas at 60 arbitrary units; auxiliary gas at 15 arbitrary units; and capillary sprayer voltages at 3,500 V and −2,500 V for positive and negative polarities, respectively. (1) MS: polarity, positive; orbitrap resolution, 30,000; mass range, *m*/*z* 120–1,200. (2) MS/MS: polarity, positive; orbitrap resolution, 15,000; mass range, *m*/*z* 120–1,200 through a data-dependent cycle time algorithm (time between master scan, 1 s); collision energy type, normalized; collision energy, 10, 30 and 50%; datatype, centroid. One QC sample was analyzed for every 10 sample solutions to account for variations in detection sensitivity during the analysis.

### Data pretreatment and chemometric analysis

#### Multivariate analysis

Peaks obtained from LC-MS were selected based on their retention times (0.5–17 min) and area values (>10^6^). Data correction was performed using blank and QC samples, with alignment achieved using retention time and *m*/*z* information. Compound Discoverer 3.3 (Thermo Fisher Scientific) was used for data acquisition.

For the multivariate analysis, the peak area values of each sample were divided by that of the internal standard. Principal component analysis (PCA) was conducted using all detected peaks. Data were normalized as z-scores and visualized using the “Principal Components” platform in JMP 17.2.0 (SAS Institute). Peak annotation and hierarchical cluster analysis (HCA) were conducted for compounds detected in *U. rhynchophylla*. Reference standards were diluted in 80% (v/v) methanol, and peaks were annotated based on their *m*/*z* values, estimated structural formulas, MS spectra, and retention times. Mean values were calculated for each sample, followed by z-scoring and clustering using Ward’s method to generate a heat map. These analyses were performed using the “Hierarchical Cluster” platform in JMP.

#### Quantitative analysis

Alkaloid quantification was performed using the internal standard method with peaks obtained from the LC-MS analysis. Peak confirmation was conducted using FreeStyle 1.8 (Thermo Fisher Scientific). Calibration curves were constructed by dividing the peak area values by the internal standard peak area and comparing them with reference standard concentrations. Each compound was dissolved in 80% (v/v) methanol and serially diluted. Calibration curves were prepared with 1 mM lidocaine added to each dilution.

Calibration curves for RC, IRC, CX, and ICX were generated using data from six different concentrations, whereas those for HTI, HTE, and GME used five different concentrations. All curves passed through the origin, assuming compound purity was 100%. Quantitative values were calculated using TraceFinder 5.1 (Thermo Fisher Scientific). Concentrations per dry weight were calculated using Excel ver.2409 (Microsoft), and the results were expressed as percentages. Graphing was performed to present the data.

## Results

### Establishment of a sterile plant culture system

The mature plants of *U. rhynchophylla* used in this experiment were grown on a slope adjacent to a valley (Supplementary Figure S1). The current-year branches which are used as raw materials in crude drugs, were soft and non-woody (Supplementary Figure S2). The seeds collected from these trees were subjected to germination under sterile conditions. To establish the sterile culture system, we compared 0.8% agar and 0.2% gellan gum as gelling agents. These solid media showed no significant differences on germination; however, the gellan gum medium became unstable, causing vitrification of the plants during subsequent growth processes. Therefore, agar was selected for the present study.

**Figure 1:**
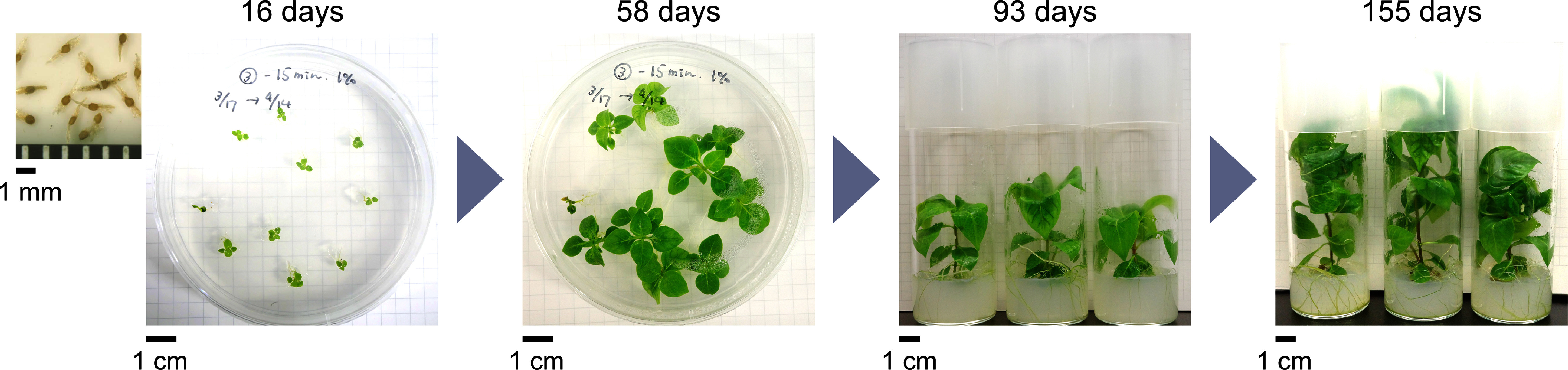
Figure 1. Sterile in vitro culture system of *U. rhynchophylla*. Seeds were sown onto φ90 mm petri dishes containing solid media. After two months of cultivation, seedlings were transferred into φ40 mm glass tubes. The plants exhibited good growth. Hooks were not formed. Refer to the scale bar for the scale of photographs. The days indicated refer to the number of days after germination.

The seeds germinated 12–14 days after seeding. Two months after germination, the body mass of each plant was approximately 3 mg dry weight. Plant mass steadily increased to 15 mg after 3 months and exceeded 20 mg after 10 months. The cultured plants exhibited progressive enlargement over time. There was minimal seedling mortality after germination, and the plants exhibited good growth (Figure 1). This trend persisted even after subculturing. However, characteristic hooks were not formed. Therefore, we expected stems to have metabolic properties similar to those of hooks from mature plants.

### Evaluation of sterile plant culture as a model of biosynthetic studies of *Uncaria* MIAs

**Figure 2:**
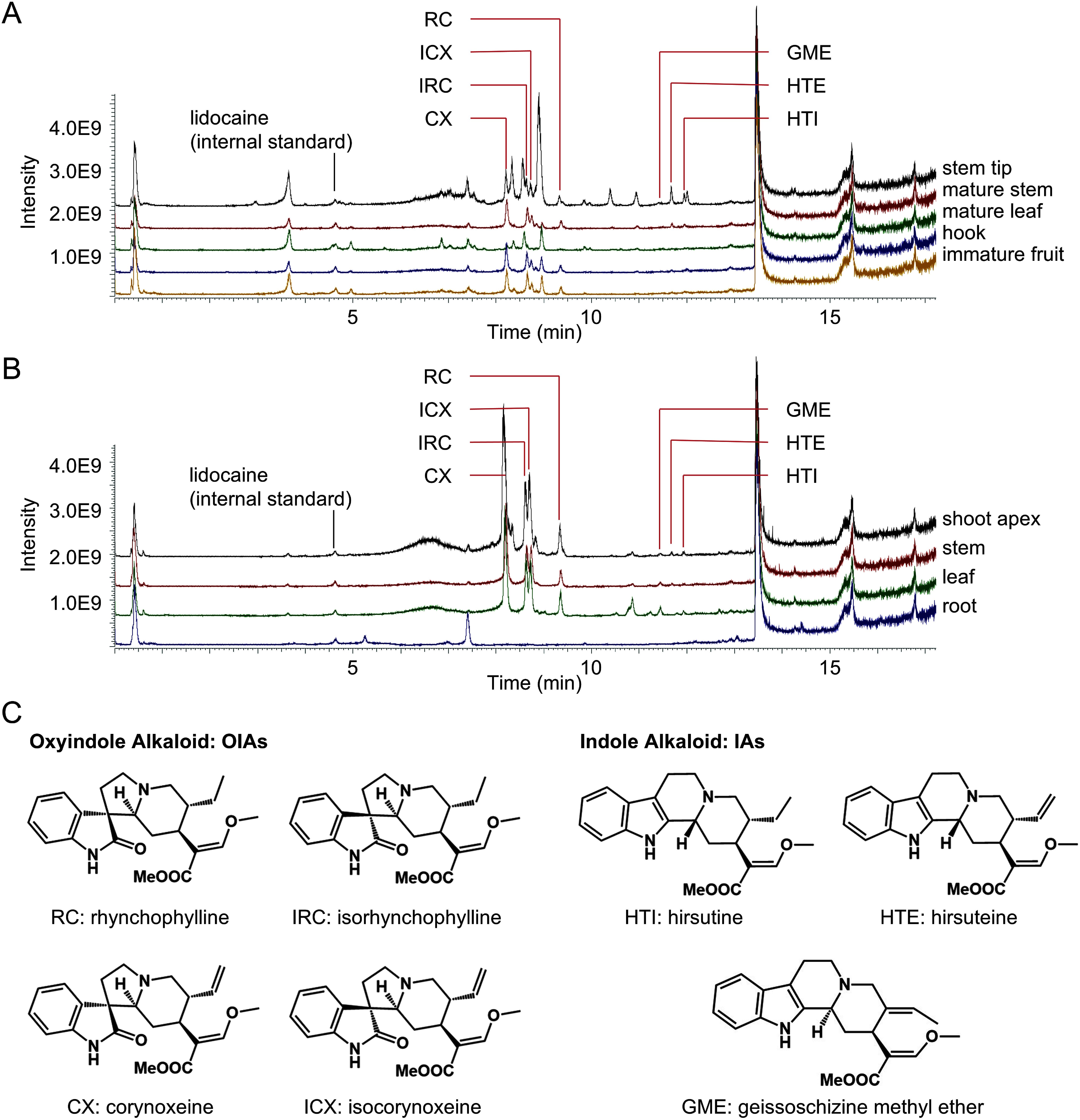
Figure 2. Comparison of compound distribution patterns in different plant tissues. A. TICC of mature plants: The TICC shows compound distribution in the stem tip, mature stem, mature leaf, hook, and immature fruit. The TICC patterns of the mature stem, hook, and immature fruit were similar. B. TICC of cultured plants at 10 months after germination: The TICC shows compound distribution in the shoot apex, stem, leaf, and root of the cultured plants. The patterns of the shoot apex, stem, and leaf were similar, while the root exhibited a distinct pattern. C. Seven representative MIAs present in *U. rhynchophylla*: The seven representative compounds were quantified and categorized into four oxindole alkaloids (OIAs) (RC, IRC, CX, and ICX) and three indole alkaloids (IAs) (HTI, HTE, and GME), based on their chemical structures.

Differences in the metabolic profiles of mature plants and sterile cultures across different tissues or developmental stages were evaluated. To provide an overview of the patterns of major compound distribution in different plant tissues, we initially compared the total ion current chromatograms (TICC) obtained from LC-MS analysis (Figure 2A, B). Peaks corresponding to the seven quantified compounds (Figure 2C) were detectable in most samples and accumulated in the stem tips of mature plants or the shoot apex of cultured plants.

In mature plants, the TICC patterns of the mature stem, hook, and immature fruit were similar (Figure 2A). This trend was inconsistent with a previous report that fruits exhibited distinct metabolite patterns compared to stems or hooks (Zhang et al. 2017). This discrepancy might be due to differences in fruit ripeness, potentially because the authors analyzed mature fruits harvested in October, while immature fruits were collected in July for the present study. In cultured plants, the patterns of the shoot apex, stem, and leaf were similar, whereas the roots exhibited a different pattern (Figure 2B). The TICC patterns showed distinct characteristics for each sample; however, the patterns of mature plant stem and hook, as well as those of cultured plant stem, were similar.

**Figure 3:**
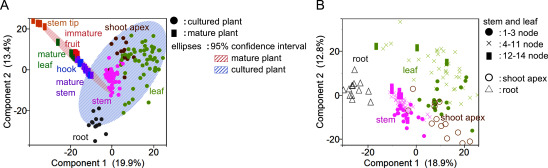
Figure 3. PCA of compound profiles in different plant tissues. A. PCA of mature and cultured plants at 10 and 12 months after germination: The score plot clearly indicates distinct separation between the compound profiles of mature (red ellipse) and cultured (blue ellipse) plants. However, the plots of the mature plant’s stem (purple) and hook (blue) were closely located to the cultured plant’s stem (pink). B. PCA of cultured plants at 10 and 12 months after germination: The score plot clearly separated shoot apex (brown), leaf (lime green), stem (pink), and root (gray) samples. Stems and leaves collected from the top part (1–3 nodes; circle mark) clustered near the shoot apex, whereas those from the bottom part (12–14 nodes; square mark) clustered near the roots, respectively.

The constituents in mature and cultured plants were further analyzed using PCA. Untargeted metabolome analysis with a relatively high-intensity threshold detected 812 peaks from the raw data. These peaks, estimated to include alkaloids, flavonoids, and sterols, were subjected to PCA. In the score plot of the PCA model, which included all samples from both mature and cultured plants, the 95% confidence ellipses for each sample were clearly separated (Figure 3A). As expected, stems and hooks from mature plants plotted close to the stems of the cultured plants. The LC-MS/MS-based untargeted metabolomics analysis demonstrated that stems in cultured plants have a good similarity in metabolite profile to the stems and hooks of mature plants, both of which are used for medicinal purposes.

### Variation in *Uncaria* MIAs based on plant tissue and developmental stage

**Figure 4:**
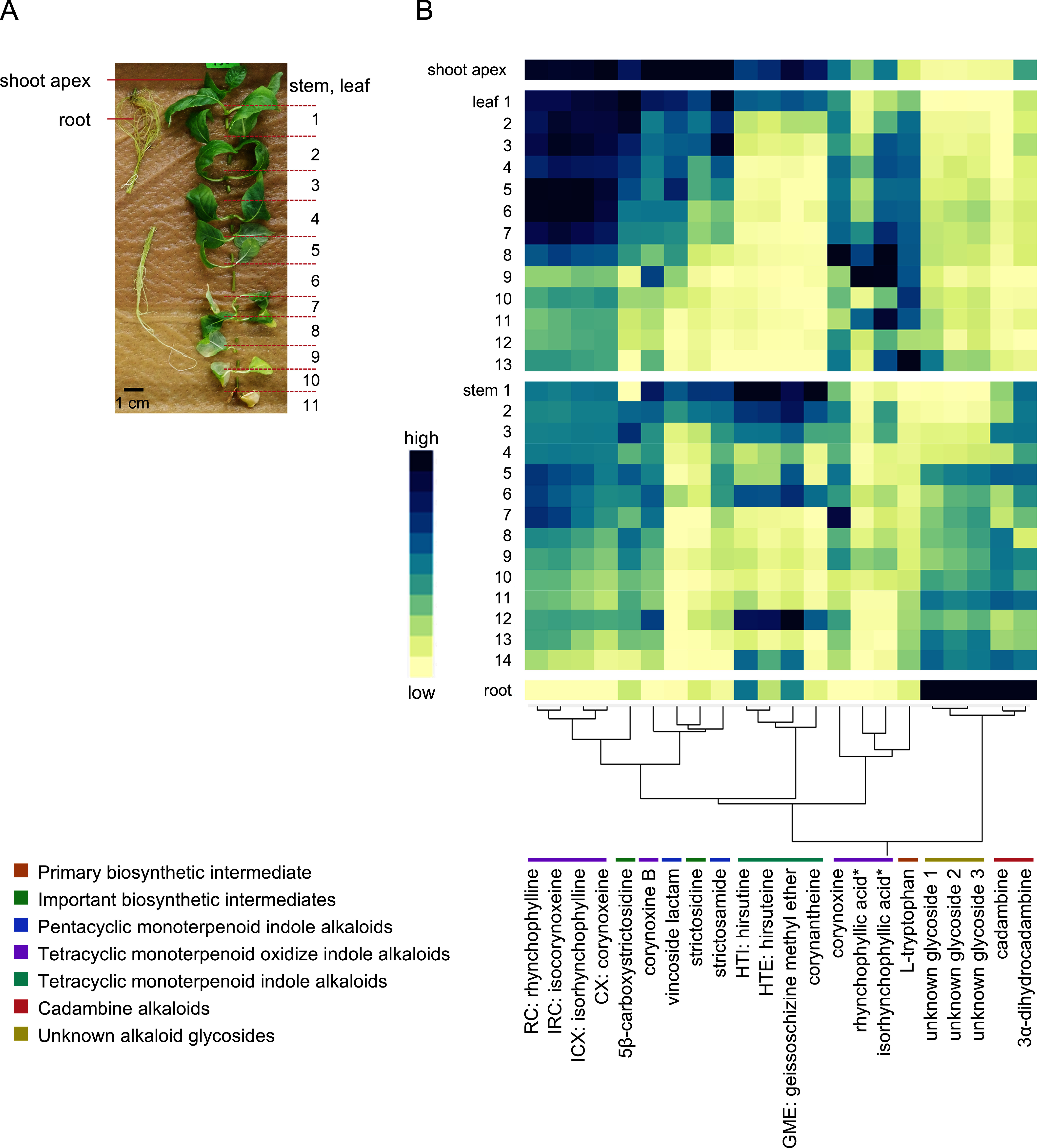
Figure 4. HCA of compound profiles in different plant tissues. A. The photograph provides a numbered reference for the cultured plant of 10 months after germination. Refer to the scale bar for the scale of photograph. B. The heatmap represents the HCA results for the 22 compounds in cultured plants of 10 and 12 months after germination. The Y-axis shows plant tissues, and the X-axis shows clustered compounds, each differentiated by color based on their characteristics or structure. The color spectrum indicates compound accumulation, with yellow indicating low accumulation and blue indicating high accumulation. Clustering revealed distinct groups: cadambine alkaloids and unknown alkaloid glycosides accumulated in the roots and formed independent clusters. Other compounds formed five clusters. For example, tetracyclic monoterpenoid oxidize indole alkaloids (RC, IRC, CX, and ICX) were concentrated in the shoot apex and upper side of the leaves, while other tetracyclic monoterpenoid indole alkaloids (corynantheine, HTI, HTE, and GME) were concentrated in the upper side of the stems, shoot apex, and roots. Similarly, other compound clusters were associated with specific plant tissues.

Next, we focused on the profiles of *Uncaria* MIAs in cultured plants to investigate how their accumulations differ depending on the tissue part or developmental stage. When a PCA was conducted using only samples from cultured plants, the score plot clearly separated the shoot apex, leaf, stem, and root samples (Figure 3B). Stems and leaves collected from the top part (1–3 nodes) clustered near the shoot apex, whereas those from the bottom part (12–14 nodes) tended to cluster near the samples from roots, respectively (Figures 3B, 4A).

To further evaluate the potential of *U. rhynchophylla* cultured plants to synthesize bioactive constituents, including MIAs, the similarity and homogeneity of the relative concentrations of selected metabolites among plant parts were explored using HCA. We selected 22 annotated compounds previously detected in *U. rhynchophylla* (Zhang et al. 2023) to generate a heat map (Table 1, Figure 4A, B). Based on clustering analysis, cadambine alkaloids, including cadambine and 3α-dihydrocadambine, as well as unknown alkaloid glycosides, were distinguished from other parameters and formed independent clusters. These compounds were confirmed to accumulate in the roots. The remaining parameters were divided into five clusters.

**Table 1:** Table 1. Annotated compounds in *U. rhynchophylla*.

		Molecular formula	Monoisotopic mass [M+H]^+^	Detected mass [M+H]^+^	Mass difference (ppm)	Retention time (min)
Primary biosynthetic intermediate						
1	L-tryptophan	C_11_H_12_N_2_O_2_	205.0972	205.0971	−0.23	2.39
Important biosynthetic intermediates (β-carboline alkaloids)						
2	strictosidine	C_27_H_34_N_2_O_9_	531.2337	531.2332	−1.04	8.36
3	5β-carboxystrictosidine	C_28_H_34_N_2_O_11_	575.2235	575.2228	−1.32	7.44
Pentacyclic monoterpenoid indole alkaloids						
4	vincoside lactam	C_26_H_30_N_2_O_8_	499.2075	499.2065	−2.02	12.01
5	strictosamide	C_26_H_30_N_2_O_8_	499.2075	499.2068	−1.47	11.34
Tetracyclic monoterpenoid oxidize indole alkaloids						
6	RC: rhynchophylline	C_22_H_28_N_2_O_4_	385.2122	385.2121	−0.15	9.29
7	IRC: isorhynchophylline	C_22_H_28_N_2_O_4_	385.2122	385.2122	−0.07	8.69
8	CX: corynoxeine	C_22_H_26_N_2_O_4_	383.1965	383.1965	−0.16	8.21
9	ICX: isocorynoxeine	C_22_H_26_N_2_O_4_	383.1965	383.1961	−1.12	8.18
10	corynoxine B	C_22_H_28_N_2_O_4_	385.2122	385.2117	−1.25	8.87
11	corynoxine	C_22_H_28_N_2_O_4_	385.2122	385.2120	−0.54	8.78
12	rhynchophyllic acid*	C_21_H_26_N_2_O_4_	371.1965	371.1970	1.15	5.99
13	isorhynchophyllic acid*	C_21_H_26_N_2_O_4_	371.1965	371.1972	1.72	7.50
Tetracyclic monoterpenoid indole alkaloids						
14	HTI: hirsutine	C_22_H_28_N_2_O_3_	369.2173	369.2170	−0.78	11.91
15	HTE: hirsuteine	C_22_H_26_N_2_O_3_	367.2016	367.2015	−0.30	11.64
16	corynantheine	C_22_H_26_N_2_O_3_	367.2016	367.2014	−0.72	10.33
17	GME: geissoschizine methyl ether	C_22_H_26_N_2_O_3_	367.2016	367.2015	−0.39	11.43
Cadambine alkaloids						
18	cadambine	C_27_H_32_N_2_O_10_	545.2130	545.2128	−0.37	6.99
19	3α-dihydrocadambine	C_27_H_34_N_2_O_10_	547.2286	547.2283	−0.64	7.41
Unknown alkaloid glycosides						
20	unknown glycoside 1	C_27_H_34_N_2_O_11_	563.2235	563.2225	−1.78	5.25
21	unknown glycoside 2	C_27_H_34_N_2_O_11_	563.2235	563.2260	4.40	6.81
22	unknown glycoside 3	C_27_H_34_N_2_O_11_	563.2235	563.2252	2.88	6.96

Peak annotation was conducted for compounds known to be present in *U. rhynchophylla* (Zhang et al. 2023) based on *m*/*z* values, estimated structural formulas, MS spectra, and retention times. Among the 22 annotated compounds, Nos. 1–11 and 14–19 were identified using reference standards, while compounds Nos. 12, 13 (indicated with * in the table), and 20–22 were estimated.

Among the tetracyclic monoterpenoid oxidize indole alkaloids RC, IRC, CX, and ICX accumulated in the shoot apex and upper leaves. Other tetracyclic monoterpenoid indole alkaloids, such as corynantheine, HTI, HTE, and GME, accumulated in the upper side of the stems, shoot apex, and roots. Important biosynthetic intermediates, such as strictosidine, pentacyclic monoterpenoid indole alkaloids, vincoside lactam, and strictosamide, accumulated specifically in the shoot apex. Among tetracyclic monoterpenoid oxidize indole alkaloids, rhynchophyllic acid, isorhynchophyllic acid, and the primary biosynthetic intermediate L-tryptophan accumulated on the lower side of the leaves.

Finally, we compared the content (%) of representative oxyindole alkaloids (OIAs), including RC, IRC, CX, and ICX, as well as indole alkaloids (IAs), including HTI, HTE, and GME, in samples from different plant parts and developmental stages of *U. rhynchophylla*. Calibration curves showed good linearity for all seven compounds (Supplementary Table S1).

The collection of mature plants was conducted in July because the highest content of MIAs was reported during this month (Kawazoe et al. 1993). Mature plants contained 0.04–0.92% of OIAs and 0–0.27% IAs, whereas cultured plants contained 0.03–3.89% OIAs and 0–0.08% IAs. OIAs were more abundant than IAs in all the plant parts. OIAs were more abundant in cultured plants than in mature plants, whereas IAs were more abundant in mature plants than in cultured plants. Among the OIAs, CX had the highest content, while HTE had the highest content among the IAs (Supplementary Tables S2, S3). Uncaria Hook has different compositions depending on its habitat, with one type rich in RC and the other rich in HTI and GME (Shi et al. 2012). Uncaria hooks derived from *U. rhynchophylla* in Japan have been reported to predominantly contain RC, which is found to be more prevalent than HTI (Mikage et al. 2008). Uncaria Hook is processed from stems with the hooks of mature plants, and in the present study, the results for the hooks and stems of the mature plants also showed that RC ranged from 0.03–0.09%, and HTI were 0–0.04% (Supplementary Table S2), confirming that RC is indeed predominant. This aligns with the trends observed in previous studies.

**Figure 5:**
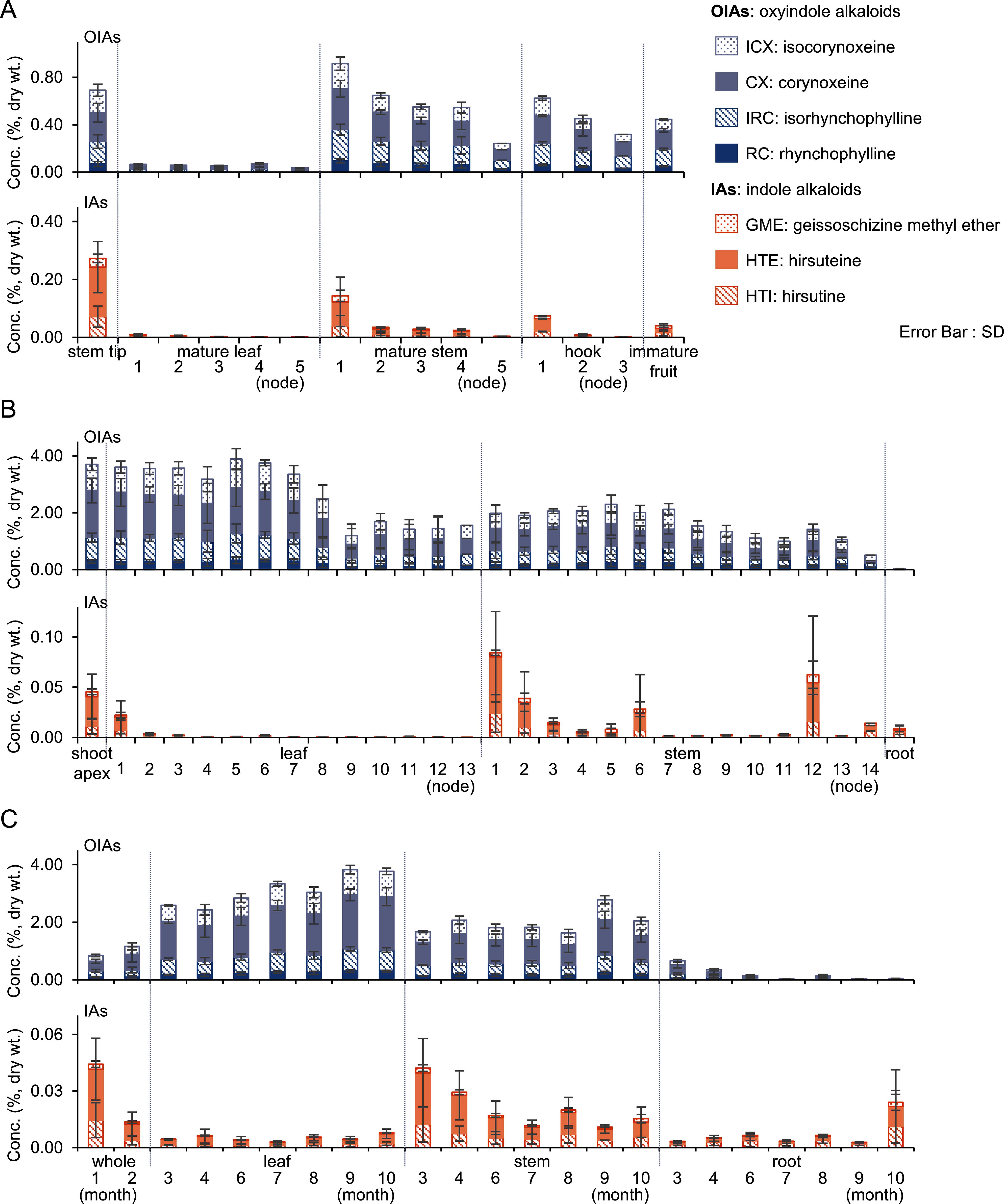
Figure 5. Comparison of MIA contents (%) in different plant parts and developmental stages of *U. rhynchophylla*. The figure is divided into two sections: the upper section shows the content of OIAs (RC, IRC, CX, and ICX), and the lower section shows the content of IAs (HTI, HTE, and GME). Stacked bar graphs with error bars (SD) are used for visualization. A. Contents in mature plants by plant tissues: OIAs and IAs were found in high amounts in the stem tip, upper side of the hook, and mature stem. B. Contents in cultured plants of 10 and 12 months after germination by plant tissues: OIAs and IAs were found in high amounts in the shoot apex, upper leaves, and stems. C. Contents in cultured plants of 1–10 months after germination by plant parts and developmental stages: The content of OIAs in leaves increased with developmental stage, while no significant trend was observed for other plant parts or IAs.

In mature plants, high amounts of OIAs and IAs were found at the stem tip, upper side of the hook, and mature stems (Figure 5A). In the cultured plants, high amounts of OIAs and IAs were observed in the shoot apex and the upper side of the leaf and stem (Figure 5B). Previous studies have reported that OIAs are primarily present in the aboveground parts, whereas IAs are primarily present in the underground parts (Yamanaka et al. 1983). However, the present study confirmed that IAs accumulated in the shoot apex rather than in the roots. The difference in developmental stages between this study and previous studies using mature plants may have influenced the results. Additionally, a previous study reported that RC and IRC are highly concentrated in the leaves and stems, whereas HTI is highly concentrated in the roots of cultured plants derived from hooks (Taniguchi and Kawamura 2016). Although the RC and IRC contents obtained from cultured plants in this study were lower than those in a previous study, the trends in the plant parts were similar. This difference in content may be attributed to individual variations or the influence of growing cultured plants from seeds. Content variation by developmental stage using cultured plants is shown in Figure 5C. The content of OIAs in the leaves showed an increasing trend according to the developmental stage; however, there were no significant trends for other plant parts and IAs.

## Discussion

In this study, we successfully established a sterile plant culture system for *U. rhynchophylla* and aimed to contribute to the elucidation as a model of the biosynthetic pathways of *Uncaria* MIAs. Figure 3A shows the proximity of the mature stem and hook in mature plants, as well as the stem in cultured plants, demonstrating that *U. rhynchophylla* can consistently produce *Uncaria* alkaloids under sterile conditions. This finding highlights the potential of cultured plants as a reliable model system for studying MIA biosynthetic pathways and ensuring quality control of medicinal Uncaria Hook. This model system would contribute to the fundamental research by enhancing our understanding of the biosynthetic mechanisms and facilitating applications such as metabolic controlling of the contents of bioactive compounds in Uncaria Hook.

Furthermore, distinct characteristics in the accumulation of key alkaloids across different plant parts were confirmed through the analysis of cultured plants, as shown in Figures 4B and 5B, C. The high accumulation of RC and HTI, which are specified in the Japanese Pharmacopoeia, in the shoot apex and the upper portions of the stem and leaf suggests that biosynthesis is actively occurring in these regions and indicates their important role in biosynthesis. These observations underscore the utility of the sterile culture system for advancing our understanding of the biosynthetic mechanisms underlying MIA production in *U. rhynchophylla*. MIAs in *Catharanthus roseus* are synthesized via multi-step enzymatic reactions in different types of cells in the leaves, suggesting the existence of regulatory mechanisms linked to development and growth (Guedes et al. 2024; Uzaki et al. 2022, 2024). While it is challenging to use woody plants as research materials, the use of sterile plant cultures may simplify the system to investigate MIA biosynthesis in *Uncaria* plants in further studies.

Additionally, as shown in Figure 5A, C, both OIAs and IAs are present in the stem and hook. Notably, the stem exhibited minimal variation in alkaloid content over time. Previous studies have focused on the selection of optimal medicinal parts of Uncaria Hook (Mikage and Endo 2008). Based on the present findings, the hook and its surrounding stem tissues, which are currently used in medicinal applications, demonstrate consistent quality and stability, reinforcing their suitability as raw materials for crude drug production from a quality control perspective.

## Abbreviations

CX: corynoxeine
GME: geissoschizine methyl ether
HCA: hierarchical cluster analysis
HTE: hirsuteine
HTI: hirsutine
IAs: indole alkaloids
ICX: isocorynoxeine
IRC: isorhynchophylline
MIAs: monoterpenoid indole alkaloids
OIAs: oxyindole alkaloids
PCA: principal component analysis
RC: rhynchophylline
TICC: total ion current chromatograms

## Description of supplementary files

Supplementary Figure S1. Area surrounding the native habitat of *U. rhynchophylla*.

Supplementary Figure S2. Details of sampling methods.

Supplementary Table S1. Calibration curve details for the seven analyzed compounds: RC, IRC, CX, ICX, HTI, HTE, and GME.

Supplementary Table S2. Content (%) of the MIAs in various plant parts of mature plant and cultured plants at 10 and 12 months after germination.

Supplementary Table S3. Content (%) of MIAs across different plant parts and developmental stages of cultured plants at 1–10 months after germination.
