# Supplementary material for: A sterile plant culture system of Uncaria rhynchophylla as a biosynthetic model of monoterpenoid indole alkaloids
Source: Plant Biotechnol (Tokyo). 2025 Jun 25;42(2):145–54. doi: 10.5511/plantbiotechnology.25.0218a (PMC12235436; doi:10.5511/plantbiotechnology.25.0218a)
Supplement: Supplementary Data [file plantbiotechnology-42-2-25.0218a-s001.pdf]

Supplementary Figure S1 Area surrounding the native habitat of *U. rhynchophylla*.

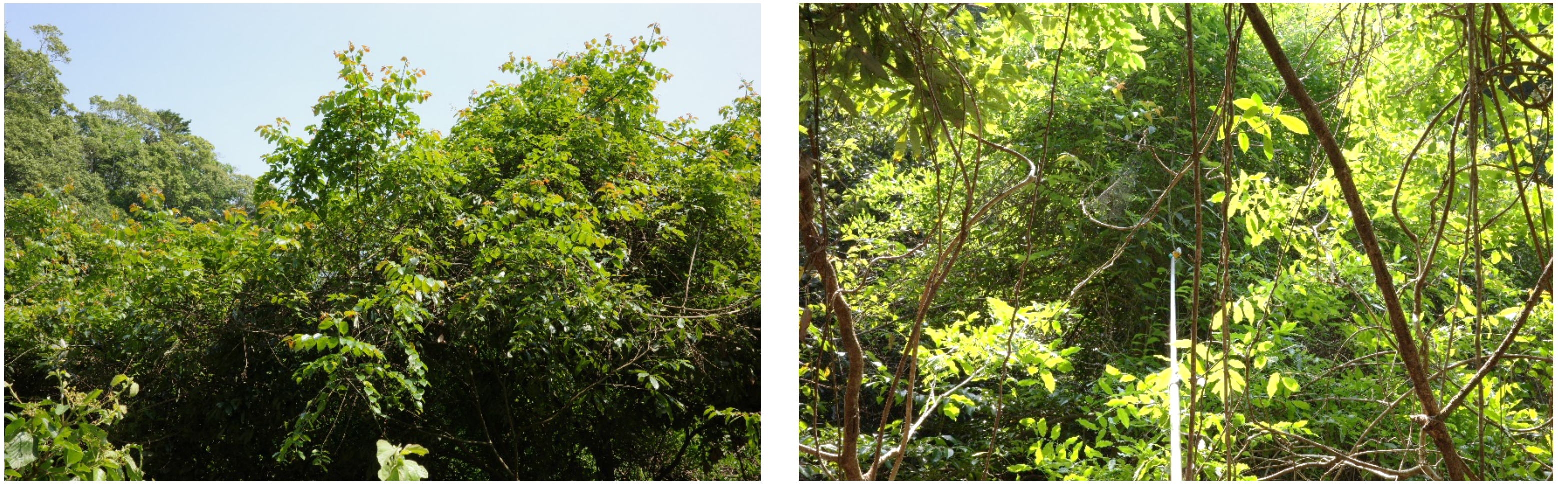

Supplementary Figure S2 Details of sampling methods.

A: The aerial parts of mature plants were separated into five tissues: stem tip, leaf, stem, hook, and immature fruit (the areas enclosed by circles were sampled). Leaves, stems and hooks at each node were sequentially numbered from the apical end of the plant stem. **B:** The seedlings of 1 and 2 month after germination were collected as whole plant material (N = 6 and 10). The photo was taken 2 months after germination. **C:** The plants of 3–10 months after germination were separated into leaf, stem, and root tissues (N = 2–9). The photo was taken 10 months after germination. **D:** The plants of 10 and 12 months after germination (N = 6), tissues were further divided into shoot apex, leaf, stem, and root. Leaves and stems at each node were sequentially numbered from the apical end of the plant stem. The photo was taken 10 months after germination.

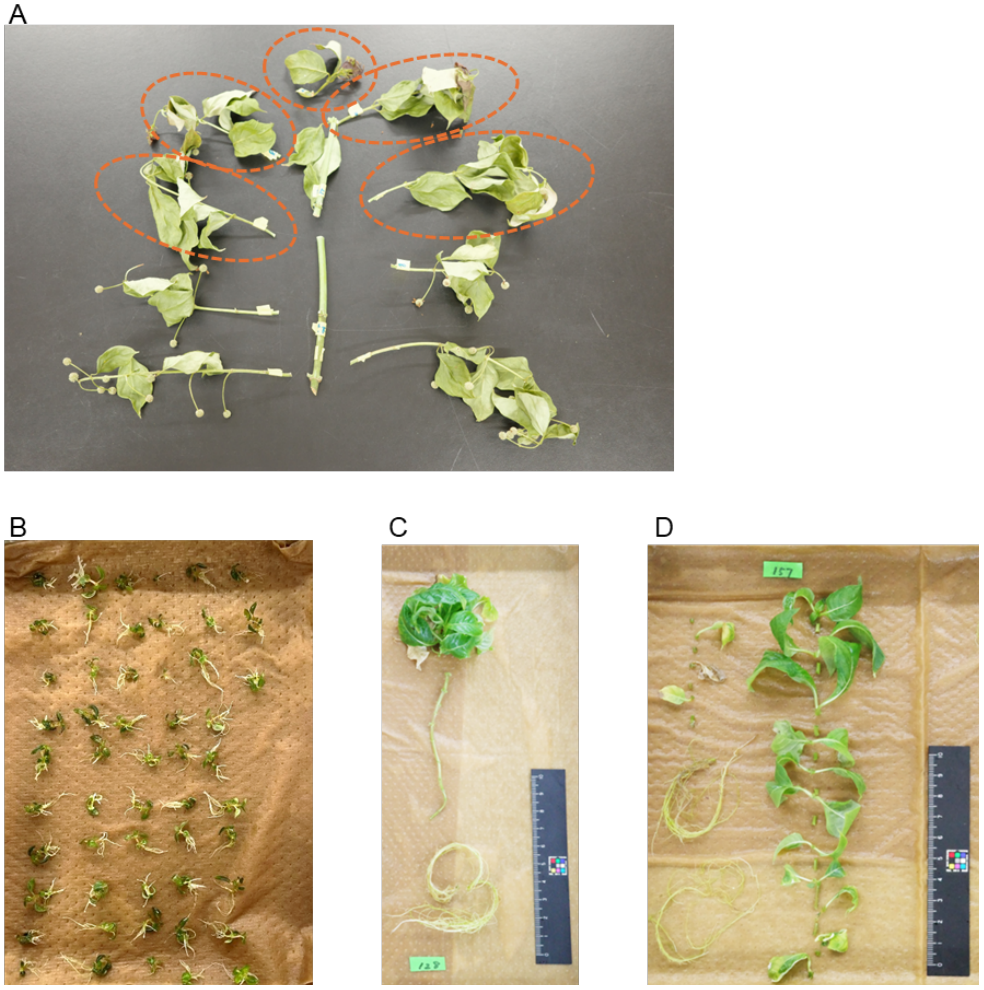

Supplementary Table S1. Calibration curve details for the seven analyzed compounds: RC, IRC, CX, ICX, HTI, HTE, and GME.

	Injected solution (ng/mL)						Figure 5A,B		Figure 5C	
	1	2	3	4	5	6	Calibration curve	R^2	Calibration curve	R^2
RC: rhynchophylline	5000.00	2500.00	1000.00	500.00	50.00	5.00	$y = 5.426e^{-3}x$	0.9998	$y = 5.000e^{-3}x$	0.9986
IRC: isorhynchophylline	5040.00	2520.00	1008.00	504.00	50.40	5.04	$y = 4.888e^{-3}x$	0.9990	$y = 4.386e^{-3}x$	0.9998
CX: corynoxeine	5035.00	2517.50	1007.00	503.50	50.35	5.04	$y = 4.839e^{-3}x$	0.9992	$y = 4.642e^{-3}x$	0.9996
ICX: isocorynoxeine	5010.00	2505.00	1002.00	501.00	50.10	5.01	$y = 3.450e^{-3}x$	0.9991	$y = 3.324e^{-3}x$	0.9999
HTI: hirsutine	502.50	201.00	100.50	50.25	10.05	-	$y = 1.010e^{-2}x$	0.9992	$y = 1.034e^{-2}x$	0.9998
HTE: hirsuteine	212.00	84.80	42.40	21.20	4.24	-	$y = 6.448e^{-3}x$	0.9995	$y = 6.846e^{-3}x$	0.9998
GME: geissoschizine methyl ether	287.00	114.80	57.40	28.70	5.74	-	$y = 7.698e^{-3}x$	0.9999	$y = 8.231e^{-3}x$	0.9993

Supplementary Table S2. Content (%) of the MIAs in various plant parts of mature plant and cultured plants at 10 and 12 months after germination.

			OIAs								IAs							
		N	RC: rhynchophylline(%)		IRC: isorhynchophylline(%)		CX: corynoxeine(%)		ICX: isocorynoxeine(%)		HTI: hirsutine(%)		HTE: hirsuteine(%)		GME: geissoschizine methyl ether(%)		OIAs(%)	IAs(%)
			Mean	SD	Mean	SD	Mean	SD	Mean	SD	Mean	SD	Mean	SD	Mean	SD	Sum	Sum
mature plant	stem tip	5	0.0688	0.0212	0.1891	0.0572	0.2437	0.0791	0.1897	0.0505	0.0717	0.0363	0.1710	0.0882	0.0303	0.0146	0.6912	0.2730
	mature leaf1	4	0.0058	0.0017	0.0209	0.0051	0.0245	0.0093	0.0141	0.0047	0.0033	0.0020	0.0052	0.0040	0.0010	0.0007	0.0653	0.0095
	mature leaf2	3	0.0051	0.0019	0.0195	0.0060	0.0217	0.0065	0.0112	0.0037	0.0022	0.0015	0.0029	0.0021	0.0006	0.0004	0.0575	0.0057
	mature leaf3	3	0.0043	0.0019	0.0179	0.0062	0.0214	0.0074	0.0092	0.0040	0.0011	0.0008	0.0014	0.0011	0.0003	0.0002	0.0528	0.0027
	mature leaf4	2	0.0055	0.0031	0.0242	0.0134	0.0282	0.0150	0.0119	0.0070	0.0007	0.0001	0.0008	0.0002	0.0002	0.0000	0.0698	0.0018
	mature leaf5	1	0.0029	-	0.0130	-	0.0159	-	0.0059	-	0.0004	-	0.0004	-	0.0001	-	0.0377	0.0008
	mature stem1	4	0.0938	0.0155	0.2649	0.0463	0.3445	0.0704	0.2124	0.0560	0.0389	0.0365	0.0840	0.0851	0.0206	0.0191	0.9157	0.1436
	mature stem2	3	0.0674	0.0188	0.1931	0.0323	0.2446	0.0186	0.1418	0.0217	0.0106	0.0046	0.0189	0.0078	0.0056	0.0026	0.6470	0.0351
	mature stem3	3	0.0582	0.0168	0.1648	0.0367	0.2126	0.0271	0.1160	0.0231	0.0093	0.0055	0.0155	0.0101	0.0041	0.0014	0.5515	0.0289
	mature stem4	2	0.0605	0.0261	0.1655	0.0726	0.2026	0.0683	0.1168	0.0454	0.0080	0.0041	0.0124	0.0085	0.0040	0.0001	0.5455	0.0244
	mature stem5	1	0.0258	-	0.0742	-	0.0892	-	0.0519	-	0.0013	-	0.0015	-	0.0012	-	0.2411	0.0039
	hook1	2	0.0608	0.0070	0.1801	0.0156	0.2351	0.0072	0.1484	0.0176	0.0204	0.0010	0.0444	0.0014	0.0093	0.0001	0.6244	0.0741
	hook2	2	0.0464	0.0051	0.1397	0.0184	0.1707	0.0522	0.0946	0.0272	0.0026	0.0031	0.0047	0.0058	0.0011	0.0012	0.4515	0.0084
	hook3	1	0.0322	-	0.1066	-	0.1189	-	0.0607	-	0.0010	-	0.0013	-	0.0005	-	0.3183	0.0027
	immature fruit	4	0.0482	0.0029	0.1457	0.0085	0.1588	0.0147	0.0916	0.0058	0.0164	0.0113	0.0150	0.0089	0.0092	0.0062	0.4443	0.0405
cultured plant	shoot apex	13	0.2820	0.0559	0.8432	0.1550	1.6584	0.4318	0.9183	0.2210	0.0112	0.0078	0.0293	0.0225	0.0049	0.0028	3.7019	0.0454
	leaf1	6	0.2777	0.0761	0.8488	0.2331	1.5907	0.4877	0.8854	0.2101	0.0074	0.0098	0.0127	0.0164	0.0020	0.0028	3.6026	0.0221
	leaf2	5	0.2824	0.0695	0.8327	0.1191	1.5262	0.2711	0.9127	0.2050	0.0012	0.0010	0.0019	0.0011	0.0005	0.0008	3.5540	0.0037
	leaf3	6	0.2958	0.0423	0.8505	0.1163	1.4628	0.3494	0.9585	0.2290	0.0009	0.0009	0.0011	0.0010	0.0002	0.0003	3.5677	0.0022
	leaf4	5	0.2571	0.1377	0.7438	0.3836	1.3295	0.7041	0.8515	0.4398	0.0002	0.0002	0.0003	0.0003	0.0001	0.0002	3.1819	0.0006
	leaf5	5	0.3272	0.1206	0.9430	0.3335	1.6106	0.6554	1.0083	0.3733	0.0003	0.0002	0.0004	0.0004	0.0001	0.0001	3.8890	0.0008
	leaf6	5	0.3237	0.0452	0.8985	0.1183	1.5254	0.2766	1.0075	0.1021	0.0005	0.0003	0.0008	0.0013	0.0001	0.0001	3.7551	0.0014
	leaf7	5	0.2932	0.0799	0.8033	0.2026	1.3348	0.4406	0.9254	0.3037	0.0002	0.0001	0.0001	0.0001	0.0000	0.0000	3.3567	0.0004
	leaf8	5	0.2149	0.1384	0.5784	0.3570	0.9890	0.6906	0.7066	0.4866	0.0004	0.0002	0.0003	0.0002	0.0001	0.0001	2.4890	0.0008
	leaf9	5	0.0993	0.1364	0.3166	0.3192	0.4690	0.5592	0.3080	0.4139	0.0002	0.0002	0.0001	0.0001	0.0000	0.0000	1.1930	0.0004
	leaf10	3	0.1352	0.0772	0.4046	0.2340	0.6896	0.4945	0.4587	0.2858	0.0004	0.0004	0.0001	0.0001	0.0000	0.0000	1.6880	0.0005
	leaf11	3	0.1100	0.0925	0.3603	0.2340	0.5838	0.5394	0.3695	0.3379	0.0005	0.0005	0.0003	0.0004	0.0000	0.0001	1.4237	0.0008
	leaf12	2	0.1188	0.1516	0.3669	0.4521	0.5998	0.7684	0.3592	0.4582	0.0003	0.0002	0.0001	0.0000	0.0000	0.0000	1.4446	0.0004
	leaf13	1	0.1512	-	0.3950	-	0.5495	-	0.4619	-	0.0001	-	0.0000	-	0.0000	-	1.5576	0.0001
	stem1	6	0.1736	0.0931	0.4950	0.2735	0.7869	0.4841	0.5252	0.2947	0.0239	0.0187	0.0565	0.0450	0.0040	0.0025	1.9807	0.0844
	stem2	6	0.1657	0.0406	0.4815	0.1403	0.7651	0.2225	0.4932	0.0906	0.0105	0.0154	0.0244	0.0306	0.0039	0.0052	1.9055	0.0388
	stem3	6	0.1783	0.0443	0.5196	0.1218	0.8168	0.1001	0.5424	0.0816	0.0036	0.0035	0.0090	0.0066	0.0021	0.0011	2.0571	0.0147
	stem4	6	0.1878	0.0522	0.5057	0.1124	0.7953	0.2239	0.5719	0.1635	0.0011	0.0010	0.0035	0.0034	0.0010	0.0008	2.0607	0.0055
	stem5	6	0.2378	0.1227	0.5816	0.2818	0.8025	0.4087	0.6762	0.3216	0.0016	0.0017	0.0034	0.0042	0.0030	0.0054	2.2981	0.0081
	stem6	6	0.2295	0.1036	0.5230	0.1855	0.6128	0.1738	0.6408	0.2537	0.0076	0.0165	0.0171	0.0379	0.0035	0.0074	2.0061	0.0282
	stem7	5	0.2458	0.1015	0.5271	0.1832	0.6532	0.1636	0.6981	0.2005	0.0005	0.0004	0.0006	0.0004	0.0002	0.0002	2.1240	0.0013
	stem8	5	0.1665	0.0424	0.3905	0.1289	0.4959	0.2727	0.4852	0.1790	0.0008	0.0006	0.0005	0.0004	0.0003	0.0003	1.5381	0.0016
	stem9	5	0.1387	0.0501	0.3286	0.1284	0.4394	0.2670	0.4359	0.2118	0.0011	0.0008	0.0010	0.0006	0.0004	0.0004	1.3427	0.0025
	stem10	5	0.1218	0.0430	0.2796	0.0574	0.3424	0.1660	0.3635	0.1662	0.0007	0.0009	0.0006	0.0006	0.0003	0.0002	1.1073	0.0016
	stem11	4	0.1169	0.0305	0.2574	0.0436	0.2768	0.1391	0.3429	0.1245	0.0014	0.0017	0.0010	0.0009	0.0005	0.0005	0.9940	0.0030
	stem12	3	0.1286	0.0437	0.3628	0.1427	0.5128	0.3481	0.4159	0.1822	0.0159	0.0269	0.0386	0.0664	0.0079	0.0135	1.4201	0.0625
	stem13	2	0.1375	0.0541	0.2755	0.0220	0.2435	0.0517	0.3979	0.0864	0.0008	0.0005	0.0005	0.0003	0.0004	0.0001	1.0544	0.0017
	stem14	1	0.0842	-	0.1480	-	0.0919	-	0.1847	-	0.0065	-	0.0054	-	0.0023	-	0.5088	0.0141
	root	13	0.0045	0.0042	0.0121	0.0093	0.0079	0.0043	0.0051	0.0032	0.0048	0.0074	0.0025	0.0043	0.0014	0.0029	0.0297	0.0087

		RC	IRC	CX	ICX	HTI	HTE	GME	OIAs Sum	IAs Sum
mature plant	min	0.0029	0.0130	0.0159	0.0059	0.0004	0.0004	0.0001	0.0377	0.0008
	max	0.0938	0.2649	0.3445	0.2124	0.0717	0.1710	0.0303	0.9157	0.2730
cultured plant	min	0.0045	0.0121	0.0079	0.0051	0.0001	0.0000	0.0000	0.0297	0.0001
	max	0.3272	0.9430	1.6584	1.0083	0.0239	0.0565	0.0079	3.8890	0.0844

Supplementary Table S3. Content (%) of the MIAs across different plant parts and developmental stages of cultured plants at 1-10 months after germination.

		OIAs								IAs							
	N	RC: rhynchophylline(%)		IRC: isorhynchophylline(%)		CX: corynoxeine(%)		ICX: isocorynoxeine(%)		HTI: hirsutine(%)		HTE: hirsuteine(%)		GME: geissoschizine methyl ether(%)		OIAs(%)	IAs(%)
		Mean	SD	Mean	SD	Mean	SD	Mean	SD	Mean	SD	Mean	SD	Mean	SD	Sum	Sum
whole_1month	6	0.0946	0.0214	0.2152	0.0495	0.3356	0.0705	0.2017	0.0412	0.0146	0.0093	0.0270	0.0164	0.0027	0.0017	0.8471	0.0442
whole_2month	10	0.0954	0.0357	0.2463	0.0974	0.5422	0.2641	0.2773	0.1187	0.0039	0.0024	0.0086	0.0063	0.0010	0.0007	1.1612	0.0135
leaf_3month	2	0.1754	0.0131	0.5387	0.0430	1.3137	0.0676	0.5619	0.0218	0.0013	0.0002	0.0029	0.0002	0.0003	0.0000	2.5898	0.0045
leaf_4month	4	0.1718	0.0488	0.4788	0.1193	1.2247	0.3992	0.5578	0.1832	0.0015	0.0009	0.0044	0.0040	0.0004	0.0003	2.4330	0.0063
leaf_6month	9	0.2184	0.0515	0.5730	0.1113	1.4114	0.3144	0.6393	0.1491	0.0012	0.0008	0.0025	0.0021	0.0003	0.0003	2.8421	0.0041
leaf_7month	5	0.2683	0.0344	0.6931	0.0785	1.6211	0.1759	0.7554	0.0841	0.0010	0.0005	0.0018	0.0010	0.0002	0.0001	3.3379	0.0030
leaf_8month	6	0.2405	0.0543	0.6060	0.1336	1.4487	0.3588	0.7433	0.1882	0.0018	0.0008	0.0034	0.0016	0.0003	0.0002	3.0384	0.0056
leaf_9month	2	0.3025	0.0411	0.7743	0.0734	1.8748	0.2025	0.8760	0.1479	0.0018	0.0013	0.0024	0.0018	0.0004	0.0002	3.8275	0.0045
leaf_10month	5	0.2975	0.0335	0.7404	0.0794	1.8509	0.3108	0.8776	0.1113	0.0021	0.0009	0.0052	0.0025	0.0006	0.0002	3.7664	0.0079
stem_3month	2	0.1332	0.0096	0.3752	0.0193	0.7938	0.0748	0.3694	0.0342	0.0123	0.0093	0.0273	0.0183	0.0025	0.0018	1.6716	0.0421
stem_4month	4	0.1740	0.0577	0.4385	0.1213	0.9905	0.3436	0.4647	0.1429	0.0074	0.0040	0.0203	0.0130	0.0016	0.0013	2.0677	0.0293
stem_6month	9	0.1623	0.0436	0.3949	0.0987	0.8248	0.2053	0.4356	0.1167	0.0052	0.0034	0.0106	0.0089	0.0012	0.0009	1.8177	0.0171
stem_7month	5	0.1708	0.0343	0.4027	0.0984	0.8058	0.2250	0.4457	0.0901	0.0045	0.0027	0.0063	0.0038	0.0009	0.0004	1.8250	0.0116
stem_8month	6	0.1481	0.0447	0.3458	0.1038	0.7178	0.2572	0.4210	0.1223	0.0069	0.0046	0.0116	0.0081	0.0014	0.0011	1.6327	0.0200
stem_9month	2	0.2508	0.0457	0.5964	0.1187	1.2390	0.2942	0.6938	0.1415	0.0039	0.0000	0.0060	0.0022	0.0010	0.0000	2.7801	0.0109
stem_10month	5	0.1841	0.0385	0.4341	0.0910	0.9063	0.2216	0.5239	0.1230	0.0056	0.0077	0.0080	0.0080	0.0019	0.0022	2.0484	0.0154
root_3month	2	0.0570	0.0142	0.1515	0.0354	0.3056	0.1023	0.1490	0.0474	0.0016	0.0005	0.0015	0.0005	0.0003	0.0001	0.6631	0.0033
root_4month	4	0.0354	0.0125	0.0852	0.0328	0.1445	0.0663	0.0847	0.0359	0.0030	0.0019	0.0019	0.0016	0.0003	0.0002	0.3497	0.0052
root_6month	9	0.0162	0.0117	0.0403	0.0305	0.0582	0.0554	0.0340	0.0291	0.0036	0.0033	0.0024	0.0022	0.0006	0.0007	0.1486	0.0065
root_7month	5	0.0042	0.0032	0.0113	0.0087	0.0127	0.0111	0.0072	0.0059	0.0021	0.0013	0.0011	0.0010	0.0002	0.0001	0.0354	0.0034
root_8month	6	0.0167	0.0122	0.0382	0.0238	0.0567	0.0341	0.0416	0.0315	0.0041	0.0018	0.0021	0.0009	0.0003	0.0003	0.1532	0.0065
root_9month	2	0.0050	0.0010	0.0132	0.0022	0.0161	0.0021	0.0096	0.0001	0.0018	0.0007	0.0007	0.0004	0.0002	0.0001	0.0439	0.0028
root_10month	5	0.0043	0.0012	0.0158	0.0048	0.0201	0.0100	0.0080	0.0018	0.0113	0.0191	0.0106	0.0194	0.0022	0.0042	0.0481	0.0240
	min	0.0042		0.0113		0.0127		0.0072		0.0010		0.0007		0.0002		0.0354	0.0028
	max	0.3025		0.7743		1.8748		0.8776		0.0146		0.0273		0.0027		3.8275	0.0442
